# Superstructured Macroporous Carbon Rods Composed of Defective Graphitic Nanosheets for Efficient Oxygen Reduction Reaction

**DOI:** 10.1002/advs.202100120

**Published:** 2021-07-29

**Authors:** Jing Wang, Yining Yao, Chaoqi Zhang, Qiang Sun, Dan Cheng, Xiaodan Huang, Jiayou Feng, Jingjing Wan, Jin Zou, Chao Liu, Chengzhong Yu

**Affiliations:** ^1^ School of Chemistry and Molecular Engineering East China Normal University Shanghai 200241 P. R. China; ^2^ Key Laboratory of Jiangsu Province for Chemical Pollution Control and Resources Reuse School of Environmental and Biological Engineering Nanjing University of Science and Technology Nanjing 210094 P. R. China; ^3^ School of Mechanical and Mining Engineering The University of Queensland Brisbane Queensland 4072 Australia; ^4^ Centre for Microscopy and Microanalysis The University of Queensland Brisbane Queensland 4072 Australia; ^5^ Australian Institute for Bioengineering and Nanotechnology The University of Queensland Brisbane Queensland 4072 Australia

**Keywords:** defects, electrocatalysis, nanosheets, porous carbon, superstructures

## Abstract

Rationally designed carbon materials with superstructures are promising candidates in applications such as electrocatalysis. However, the synthesis of highly porous carbon superstructures with macropores and carbon defects from a simple crystalline solid remains challenging. In this work, superstructured macroporous carbon rods composed of defective graphitic nanosheets are synthesized by direct carbonization of crystalline poly tannic acid (PTA) rods as precursors. During carbonization, PTA rods with a highly ordered lamellar structure induce a spatially confined two‐step localized contraction that takes place in different dimensions and directions in each step. The unexpected contraction behavior results in the sponge‐like macroporous carbon superstructure with large surface area, high porosity, and abundant defects, thus showing a superior electrocatalytic performance with high activity and selectivity for oxygen reduction reaction. The study provides new understandings in the design of functional carbon materials with distinctive structures and applications.

## Graphical abstract

Superstructured macroporous carbon rods composed of defective graphitic nanosheets are synthesized by direct carbonization of crystalline poly tannic acid rods as a new carbon precursor. A spatially confined two‐step localized contraction mechanism is proposed for the simultaneous formation of macroporous superstructure and defects, resulting in a remarkable oxygen reduction reaction performance.

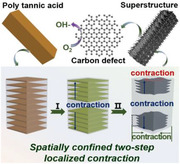

## 1 Introduction

Carbon nanomaterials with various morphologies have received extensive attention in many fields owing to their appealing physicochemical properties such as abundance, low cost, high conductivity, and tunable structures.^[^ ^1^ ^]^ Among different carbon structures, 3D carbon superstructures assembled by simple building blocks such as nanotubes, nanofibers, nanoparticles, and nanosheets have gained increasing interest due to their excellent performance in energy and environmental applications.^[^ ^2^ ^]^ In addition to the assembly of presynthesized low dimensional carbon materials as building blocks,^[^ ^3^ ^]^ an alternative strategy is direct carbonization of suitable precursors to form carbon superstructures. The precursors usually have preformed superstructures and are directly carbonized into corresponding carbon materials with dominating micro/mesopores.^[^ ^2^, ^3^ ^]^ For example, a flower‐like superstructure of polyimide nanosheets was synthesized and converted into similarly structured carbon material.^[^ ^3c^ ^]^ Xu and co‐workers^[^ ^2a^ ^]^ fabricated a spherical metal organic framework (MOF) superstructure composed of 1D nanorods for the construction of chestnut‐shell‐like carbon materials. Zinc zeolitic imidazolate framework (ZIF‐8) dodecahedron prearranged by polyvinylpyrrolidone (PVP) was also used as a precursor for the synthesis of a honeycomb‐like carbon superstructure.^[^ ^2d^ ^]^ However, there are rare reports on the synthesis of carbon superstructures by direct pyrolysis of a simple precursor such as crystalline solids.

In addition to the morphology, creation of defects in carbon nanostructures has shown great potential in tuning their electronic structure and improving their property especially as electrocatalysts.^[^ ^4^ ^]^ Currently, there are mainly two strategies for producing carbon defects: 1) removal of heteroatoms (e.g., N and Zn) during heat treatment of selected precursors,^[^ ^4a,b,e,h,p^ ^]^ and 2) removal of carbon atoms from as‐formed graphitic carbon materials (e.g., graphene, carbon, and nanotube) by physical methods such as plasma or milling treatment.^[^ ^4l,n^ ^]^ Nevertheless, the resultant defective carbon materials are mainly in the forms of solid nanoparticle, nanotube, nanosheet, and nanocage with micropore‐dominated structures.^[^ ^4c,f,g^ ^]^ There are a few reports of defective carbon materials with 3D macropores, which is beneficial for mass transport and the exposure of active sites for electrocatalysis.^[^ ^5^ ^]^ Moreover, creating defects in graphitic carbon using new strategies is of great significance, but rarely reported.

**Scheme 1:**
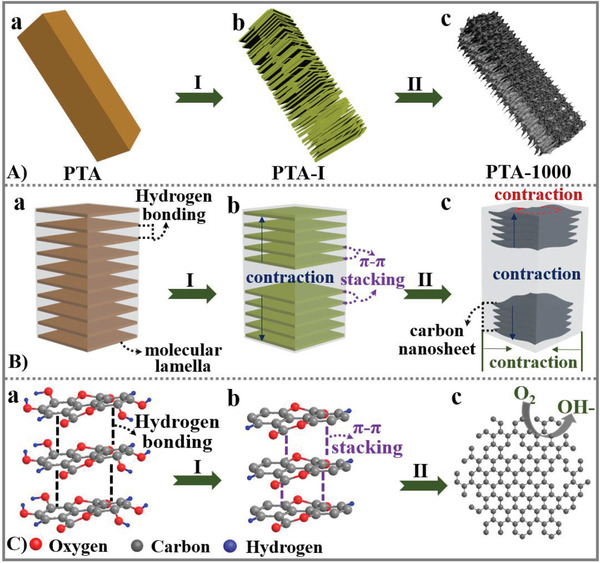
Schematic illustration of A) morphology, B) layered subunit, and C) molecular/atomic structure changes from PTA to PTA‐1000. PTA transfers into an intermediate (denoted as PTA‐I) at 250 °C (step II), which further converts into PTA‐1000 at 1000 °C (step II). The number of lamellas in one nanosheet is for illustration only.

Herein, for the first time, sponge‐like superstructured macroporous carbon rods composed of curly graphitic carbon nanosheets with abundant carbon defects are fabricated by direct carbonization of crystalline poly tannic acid (PTA) rods (**Scheme** **1**A). Different from the reported carbon superstructures that mostly inherit the morphology of precursors with homogeneous contraction, PTA rods with a highly ordered lamellar structure induce a spatially confined two‐step localized contraction during the carbonization process. The first step contraction occurs at a low carbonization temperature along the longitudinal axis of the rod, driven by the dehydration between adjacent lamellas and the in situ generated *π*–*π* stacking between adjacent molecular layers. This contraction is locally uneven, confined by the rod‐like morphology with the change in length less significant than weight loss, creating nanosheets assembled by ≈15 lamellas and inter‐nanosheet space, but along the whole rod it is relatively homogenous. The second step contraction happens at higher temperature in three dimensions caused by carbonization and graphitization, forming macroporous carbon superstructure with defects generated by in‐plane confined contraction (Scheme 1B,C). Benefiting from the structural merits, the resultant superstructure as an electrocatalyst demonstrates superior performance with high activity and selectivity for oxygen reduction reaction.

## 2 Results and Discussion

**Figure 1:**
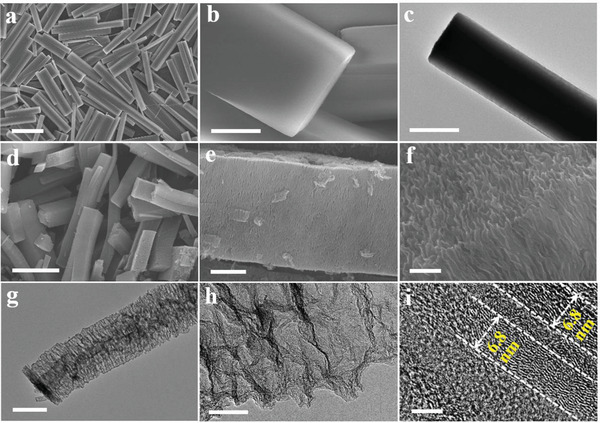
a,b) SEM and c) TEM images of PTA, d–f) SEM, and g–i) TEM images of PTA‐1000. The scale bars are a) 10 µm, b,g) 1 µm, c) 2 µm, d) 5 µm, e) 500 nm, f) 200 nm, h) 50 nm, i) and 5 nm.

Poly tannic acid (PTA) rods were prepared by a hydrothermal process using tannic acid as a precursor. Field‐emission scanning electron microscopy (SEM) images show that the PTA product has a uniform rod‐like morphology with length of 10–30 µm and width of 2–3 µm (**Figure** **1**a; Figure S1, Supporting Information). Magnified SEM (Figure 1b) and transmission electron microscopy (TEM) images (Figure 1c) show that the PTA rods have a smooth surface and solid structure.

After thermal treatment in N_2_ atmosphere at 1000 °C, PTA rods were converted into a carbon material (denoted as PTA‐1000). As shown in Figure 1d–e and Figure S2 (Supporting Information), PTA‐1000 possesses a well retained rod‐like morphology with a porous nature. The sponge‐like nanoporous framework is comprised of curly nanosheets as shown in the enlarged SEM image (Figure 1f). The sponge‐like nanoporous structure is further evidenced by TEM observations (Figure 1g). The distance between nanosheets is not uniform, estimated to be as large as 50–100 nm (Figure 1h). In the high‐resolution TEM (HRTEM) image (Figure 1i), clear lattice fringes with a lattice spacing of ≈0.35 nm are observed along the parallel direction of carbon nanosheet, suggesting a graphitized carbon structure. The thickness of carbon nanosheet is measured to be ≈6.8 nm, which is composed of ≈15 layers of graphene‐like carbon monolayers. Both SEM and TEM observations indicate that PTA‐1000 has a rod‐like superstructure assembly by curly graphitic carbon nanosheets as the building blocks with sponge‐like large pores.

**Figure 2:**
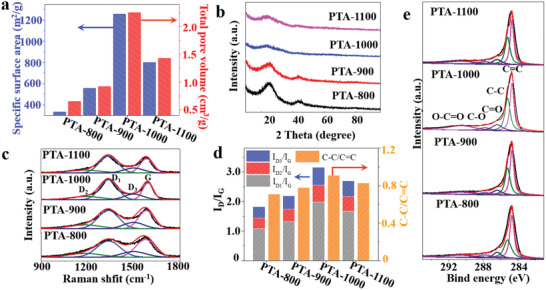
a) BET surface area and pore volume, b) XRD spectra, c) Raman spectra, d) *I*_D_/*I*_G_ and C—C/C═C ratios, and e) high‐resolution XPS spectra of C1s of PTA‐800, 900, 1000, and 1100.

To investigate the impact of carbonization temperature (*T*) on the final structure, other three temperatures (namely, 800, 900, and 1100 °C) were also selected for comparison. The resultant samples were denoted as PTA‐T (*T* = 800, 900, and 1100). As presented in SEM and TEM images (Figure S3a–f, Supporting Information), PTA‐800 and 900 exhibit a similar superstructure to PTA‐1000. However, by increasing the carbonization temperature to 1100 °C, the superstructure collapsed, forming large size graphene‐like carbon sheets (Figure S3g–I, Supporting Information). The surface area and pore structure of PTA‐T samples were examined by N_2_ sorption analysis. As shown in Figure S4 (Supporting Information), the N_2_ sorption isotherm of PTA‐1000 exhibits a typical H4 hysteresis loop in high relative pressure (*P*/*P*_0_) regions (0.9–1.0), revealing the existence of macropores.^[^ ^6^ ^]^ The pore size distribution curve of PTA‐1000 (Figure S4b, Supporting Information) further confirmed the existence of macropores with ≈100 nm, similar to the distance between carbon nanosheets observed in TEM image. Among all carbon samples, PTA‐1000 possesses the highest Brunauer–Emmett–Teller (BET) specific surface area of 1256 m^2^ g^−1^, micropore, mesopore, and total pore volume of 0.34, 1.04, and 2.25 cm^3^ g^−1^, respectively (**Figure** **2**a; Figure S4 and Table S1, Supporting Information).

To further characterize the structural difference of all PTA‐T samples, X‐ray diffraction (XRD) and Raman spectra techniques were used. In the XRD patterns of all four samples (Figure 2b), two diffraction peaks at 25.1° and 43.3° are detected, which can be assigned to (002) and (101) planes of the graphitized carbon. The peak intensity gradually reduces with increasing the temperature from 800 to 1000 °C, then increases at 1100 °C, suggesting the lowest crystallinity of PTA‐1000. The Raman spectra (Figure 2c) can be deconvoluted into four types of carbon configurations at 1336 (D_1_), 1186 (D_2_), 1500 (D_3_), and 1592 (G) cm^−1^, respectively. The D_1_ band is ascribed to the defects and disorders in the carbon lattice while the G band corresponds to the ordered sp^2^‐bonded graphite carbon.^[^ ^7^ ^]^ The D_2_ band is associated with carbon atoms around the grain boundaries of crystalline graphene, regarded as edge defects.^[^ ^7^, ^8^ ^]^ In addition, the D_3_ band is related to the amorphous sp^3^ carbon, the intensity of which is inversely related to G band.^[^ ^8^, ^9^ ^]^ The intensity ratios of *I*_D1_/*I*_G_, *I*_D2_/*I*_G_, and *I*_D3_/*I*_G_ based on peak area were shown in Figure 2d and Table S2 (Supporting Information). The values are found to increase first when the temperature is increased from 800 to 1000 °C, then decreased at 1100 °C, indicating that highest content of carbon defects is achieved at 1000 °C, in agreement with XRD results. According to previous reports,^[^ ^4a–c,i–n^ ^]^ both the D_1_‐ and D_2_‐type defects are regarded as the active sites for electrocatalytic oxygen reduction.

The carbon structure of PTA‐1000 was further analyzed by X‐ray photoelectron spectroscopy (XPS) with PTA‐800, 900, and 1100 as comparison. The XPS survey spectrum (Figure S5, Supporting Information) of PTA‐1000 shows the existence of only C and O elements without other metals or heteroatoms doped. The C 1s spectra of the four samples can be deconvoluted into five peaks at 284.7, 285.2, 286.4, 287.8, and 290.5 eV, which can be attributed to C═C (sp^2^), C—C (sp^3^), C—O, C═O, and O═C—O, respectively (Figure 2e).^[^ ^4o^ ^]^ The peak area ratio (Figure 2d) of C—C/C═C for PTA‐1000 (0.91) is higher than those of PTA‐800 (0.71), 900 (0.78), and 1100 (0.83), confirming its highest content of defective carbon. The atomic O/C ratios of PTA‐T calculated from XPS spectra are also given in Figure S6 in the Supporting Information (3.57, 3.25, 2.65, and 1.77% at 800, 900, 1000, and 1100 °C, respectively), indicating a higher degree of oxygen removal for samples treated at higher temperature. Collectively, XPS, XRD, and Raman results have shown that the ratio of defective carbon increases with carbonization temperature first and then decreases, reaching a maximum at 1000 °C. Generally, a higher graphitization degree with a lower content of defects is obtained with increased carbonization temperature. Unexpectedly, the lowest graphitization degree and highest content of defects is achieved in PTA‐1000. These results indicate that as long as the rod‐shaped structure is maintained, the graphitization process is hindered and more defects are generated at higher temperature. Therefore, the PTA with a preformed rod morphology is crucial for the formation of macroporous and defective structure during the carbonization process.

As a natural polyphenol compound, TA and its derivatives have been widely used as precursors for fabricating carbon‐based materials such as solid particles, nanotubes, nanosheets, mesoporous monolith, and hollow nanoparticles.^[^ ^10^ ^]^ Notably, our synthesized defective sponge‐like superstructure is different from reported TA‐derived carbon materials, where metal species were introduced in most studies. For example, Wang and co‐workers.^[^ ^10c^ ^]^ reported a hydrothermal synthesis of metal (Co or Fe)‐PTA coordination crystals. After carbonization, the graphitization degree of metal doped carbon materials increased (evidenced by decreased *I*_D_/*I*_G_) with increasing the temperature. Metal‐free PTA materials have also been synthesized for bioapplications, but not as carbon sources.^[^ ^10^, ^11^ ^]^ Even for carbon materials derived from other precursors (e.g., resin, metal organic framework, and covalent organic framework), usually a lower content of carbon defects was generated at higher temperature with increasing the graphitization,^[^ ^7^, ^12^ ^]^ different from our observations that the highest defective carbon content, specific surface area and pore volume at a carbonization temperature of 1000 °C.

For comparison, TA was directly carbonized at 1000 °C and generated a TA‐1000 sample. The control sample shows a dense structure with a low surface area of 89 m^2^ g^−1^ and pore volume of 0.10 cm^3^ g^−1^ (Figure S7a–c and Table S1, Supporting Information). XRD, Raman, and XPS results (Figure S7d–f, Supporting Information) indicate that TA‐1000 exhibits a higher degree of graphitization than PTA‐1000. These results further indicate that the formation of PTA rods is important to generate PTA‐1000 with a unique sponge‐like defective carbon superstructure.

**Figure 3:**
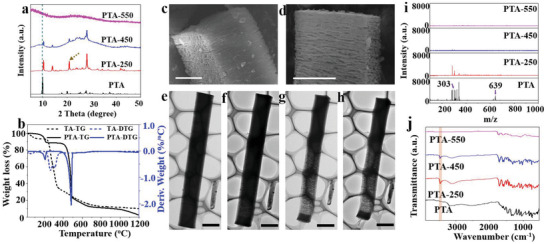
a) XRD patterns, b) TGA curves of TA and PTA, and SEM images of c) PTA‐250 and d) PTA‐450. In situ TEM images e–h) of PTA‐100, PTA‐250, PTA‐350, and PTA‐450. Scale bars are 1 µm c,d) and 2 µm e–h), and i) MALDI‐TOF MS spectra and j) FTIR spectra of PTA, PTA250‐550.

To understand the crystalline structural difference between TA and PTA, XRD characterization was carried out. As shown in Figure S8 (Supporting Information), TA possesses an amorphous structure, in accordance with literature results.^[^ ^13^ ^]^ In contrast, XRD patterns of PTA (**Figure** **3**a) show a group of diffraction peaks at 9.9°, 20.0°, and 30.2° with gradually decreased intensity, corresponding to (001), (002), and (003) diffractions of a well‐ordered lamellar structure in the PTA rod. The XRD patterns of PTA rods treated at 250, 450, and 550 °C were also recorded. After heat treatment at 250 and 450 °C, the diffraction peaks shift toward larger 2*θ* compared to PTA, indicating reduced spacing of molecular layers owing to the slight contraction. Meanwhile, the relative peak intensity is also changed with a new peak generated at 13.9°, which may be ascribed to the structural change of distorted molecular layers under heat treatment.^[^ ^14^ ^]^ Notably, another new and intense diffraction peak at ≈20.8° is also observed, which is likely attributed to the *π*–*π* stacking interaction between the lamellar PTA molecules.^[^ ^15^ ^]^ By further increasing the temperature to 450 °C, the intensities of all diffraction peaks are weakened with no position shift, which totally disappear at 550 °C due to dramatical decomposition and carbonization.

The thermogravimetric analysis (TGA) was then conducted to explore the weight loss behaviors of TA and PTA as a function of temperature. The TGA and corresponding derivative thermogravimetry (DTG) curves are displayed in Figure 3b. For TA, the weight loss of 6.5% at 120 °C is attributed to the removal of absorbed solvents. The dramatical weight loss from 230 to 700 °C is originated from the thermal decomposition and carbonization. Differently, negligible weight loss of 0.9% is observed in the TGA curve of PTA before 120 °C, while an obvious weight loss of 10.2% is found between 120 and 220 °C. This should be caused by the thermal condensation of interplane OH groups, resulting in reduced layer distance as indicated by XRD patterns. In the range of 220 to 400 °C where the *π*–*π* stacking interaction generates according to XRD observations, there is almost no weight loss for PTA. The further thermal decomposition occurs at temperature of 400–500 °C, in the range where the *π*–*π* stacking interaction starts to disappear, suggesting the in situ generated *π*–*π* stacking interaction between PTA layers increases its thermal stability. The weight loss after 500 °C is mainly attributed to the carbonization process.

Furthermore, the morphology and pore structure changes of PTA rods treated at different temperatures were studied by SEM and nitrogen sorption analysis. PTA has a solid structure and smooth surface (Figure 1b). After heat treatment, slits were observed on the surface with the slit size increasing with increased temperature from 250 to 450 °C (Figure 3c,d; Figure S9, Supporting Information). TEM was also used to understand the in situ morphology change of a typical PTA rod upon heating from room temperature to 450 °C with a heating rate of 10 °C min^−1^ (Figure 3e–h). The solid PTA rod was transformed into a porous structure at 250 °C with 2.6% of length decrease but negligible width reduction (Figure S10, Supporting Information). Considering the obvious weight loss of 11.4% at this temperature (from TGA), SEM and TEM observations indicate a localized contraction of ≈15 lamellas (evidenced from Figure 1i) into one nanosheet along the longitudinal axis of the rod. This contraction is spatially uneven because the length of the rod has little change. The localized contraction confined in a preformed crystalline rod‐like morphology leads to the slits or porous space between two adjacent nanosheets. By rising the temperature to 450 °C, a strong reduction in width of 14.3% than length of 13.7% was observed, indicating the contraction occurred both within the lamellar plane and along the longitudinal axis of the rod, causing increased pore volume as evidenced by the N_2_ isotherms studies (Figure S11, Supporting Information).

Matrix‐assisted laser desorption/ionization time of flight mass spectroscopy (MALDI‐TOF MS) and Fourier transform infrared (FTIR) spectroscopy were carried to study the chemical structure evolution. The MALDI‐MS spectrum of PTA is shown in Figure 3i. Typical peaks assigned to ellagic acid (EA, *m*/*z* = 302) and EA derivatives (*m*/*z* = 638) are found (Figure S12a,b, Supporting Information), which can be regarded as the basic building blocks for PTA assembly.^[^ ^13^ ^]^ For PTA‐250, the peak intensities were reduced, most likely due to the formation of a more condensed framework. Less or even no molecular fragments were detected at 450 and 550 °C, indicating an even more stable framework or destroyed building blocks. By comparing the FTIR spectrums of TA (Figure S13, Supporting Information) to PTA (Figure 3j), the band of O–H group shifts from ≈3341 (TA) to 3191 cm^−1^ (PTA), indicating that the PTA is assembled by EA derivatives as building blocks through intermolecular hydrogen bonding interaction.^[^ ^15^ ^]^ Compared to PTA, a new peak at higher wavenumber of 3465 cm^−1^ is generated, which may be ascribed to the formation of O—H···*π* hydrogen bond with higher bond energy,^[^ ^16^ ^]^ in accordance with the XRD results. By increasing the temperature to 450 °C, the band intensities of O–H decrease due to the further dehydration at higher temperature. For PTA‐550, only weak bands of C═O, C—O, and benzene ring are remained owing to higher degree of carbonization.

Based on the above observations, we propose a spatially confined two‐step localized contraction mechanism responsible to the formation of PTA‐1000 rods with a sponge‐like superstructure consisted of curly carbon nanosheets and abundant intrinsic carbon defects (Scheme 1). First, PTA rods with highly ordered lamellar structures are assembled by EA derivatives via intermolecular hydrogen bonding (Scheme 1Aa,Ba,Ca and Figure 3a,i,j).^[^ ^13^ ^]^ Second, in the initial stage of pyrolysis process (i.e., 250 °C), dehydration reaction (step I) takes place between the O–H groups (Figure 3j), reducing the distance and forming *π*–*π* stacking interaction between adjacent lamellas in the intermediate PTA‐I (Scheme 1Cb and Figure 3a). Due to more significant change in weight loss than the volume decrease (Figure 3b,e–g), there is a localized first‐step contraction of ≈15 lamellas into one nanosheet along the longitudinal axis of rod with the whole rod diameter almost unchanged (Figure 1i and 3c), creating space between nanosheets. This locally uneven contraction is confined by the preformed rod‐like morphology, and occurs indeed “evenly” along the entire rod, forming a porous rod consisted of nanosheets (Scheme 1Bb; Figure S11, Supporting Information). In the further pyrolysis step (step II), the second‐step contraction occurs with both obvious weight loss and volume reduction (Figure 3b,d,h). The contraction is anisotropic along longitude and short axis directions (Figure S10, Supporting Information), leading to curly nanosheets with abundant edge defects (Scheme 1Bc,Cc and Figure 2c,d), which are interconnected into the 3D sponge‐like carbon superstructure (Scheme 1Ac and Figure 1d–h). While the second step in‐plane contraction (Scheme 1Cc) is mainly responsible for creating in‐plane defects and pores especially at 1000 °C with a higher specific surface area, further increasing the temperature (e.g., 1100 °C) causes the collapse of superstructure with lower contents of defects and decreased specific surface area due to graphitization (Figure 2; Figure S4 and Table S1, Supporting Information). The preformed PTA rods with high crystallinity is essential for the localized contraction, thus TA precursors form only dense carbon monolith (Figure S7, Supporting Information).

The syntheses and formation mechanism of PTA‐1000 is significantly different from literature reports. For generating macropores, the reported strategies mostly rely on the assistance of additional templates (e.g., colloid particles,^[^ ^17^ ^]^ surfactants,^[^ ^18^ ^]^ and emulsion^[^ ^19^ ^]^). For producing defects, as mentioned before, two pathways including removal of heteroatoms (e.g., N and Zn) by heating treatment and removal of carbon atoms by physical methods (e.g., plasma and milling) were generally used. In our design, the macropores and defects were simultaneously created by directly carbonization of crystalline PTA rods through a confined two‐step localized contraction route.

**Figure 4:**
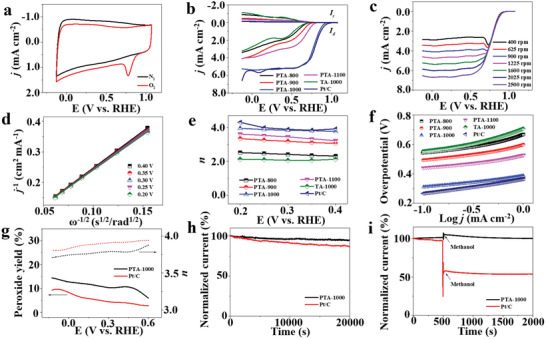
a) CV curves of PTA‐1000 in O_2_ or N_2_‐saturated 0.1 m KOH solution with a scan rate of 5 mV s^−1^. b) Rotating ring‐disk electrode (RRDE) voltammograms of PTA‐800, 900, 1000, 1100, TA‐1000, and Pt/C in O_2_‐saturated 0.1 m KOH solution at 1600 rpm. c) Linear sweep voltammetry (LSV) curves of PTA‐1000 in O_2_‐saturated 0.1 m KOH solution at different rotation rates. d) Koutecky–Levich plots of PTA‐1000 derived from the LSV curves in (c) at different potentials. e) Electron transfer number (*n*) calculated from K–L equations at different potentials. f) Tafel plots. g) Percentage of peroxide species (solid lines) and the electron transfer number (*n*) (dotted lines) of PTA‐1000 and commercial Pt/C at different potentials. h) Current–time chronoamperometric durability test of PTA‐1000 and Pt/C. i) Current–time response of PTA‐1000 and Pt/C in O_2_‐saturated 0.1 m KOH solution (0–500 s) and O_2_‐saturated 0.1 m KOH solution with methanol (500–2000 s) at a rotation rate of 1600 rpm.

Oxygen reduction reaction (ORR) is a key step that determines the performance of various next‐generation devices for energy storage and conversion, such as fuel cell, metal–air batteries, and oxygen sensors.^[^ ^20^ ^]^ Among various catalysts, defective carbons are regarded as promising candidates of Pt‐based materials. Here the electrochemical performance of PTA‐1000 was investigated by testing the ORR performance in a three‐electrode system with PTA‐800, 900, 1000, TA‐1000, and commercial Pt/C catalyst as control. The ORR activity was first evaluated by cyclic voltammetry (CV) performed in N_2_‐ and O_2_‐saturated electrolyte (0.1 m KOH) at a scan rate of 5 mV s^−1^. As shown in **Figure** **4**a, a sharp ORR peak is observed at 0.78 V (vs reversible hydrogen electrode (RHE); see Figure S14 in the Supporting Information for RHE calibration) for PTA‐1000 only in O_2_‐saturated condition, demonstrating its ORR activity. Among the studied catalysts (Figure S15, Supporting Information), PTA‐1000 exhibits a more positive reduction peak with a higher current intensity of 1.34 mA cm^−2^ than those of PTA‐800 (0.60 V vs RHE, 0.28 mA cm^−2^), 900 (0.66 V vs RHE, 0.24 mA cm^−2^), 1100 (0.70 V vs RHE, 0.32 mA cm^−2^), and TA‐1000 (0.53 V vs RHE, 0.17 mA cm^−2^), indicating the highest ORR activity of PTA‐1000.

Linear sweep voltammogram (LSV) polarization curves of different catalysts were recorded in O_2_‐saturated 0.1 m KOH solution via rotating ring‐disk electrode (RRDE) measurements at a rotation rate of 1600 rpm (Figure 4b). The ORR onset potential of PTA‐1000 is more positive than PTA‐T treated at other temperatures and TA‐1000, which is even close to that of Pt/C (Table S3, Supporting Information). The half‐wave potential (*E*_1/2_) of PTA‐1000 was determined to be 0.78 V, higher than that of PTA‐800 (0.57 V), 900 (0.63 V), 1100 (0.68 V), and TA‐1000 (0.53 V), further revealing its excellent ORR activity. Notably, the *E*_1/2_ value of PTA‐1000 is close to that of commercial Pt/C (0.80 V) and comparable to most reported defective carbon electrocatalysts (Table S4, Supporting Information).^[^ ^4^, ^21^ ^]^

The LSV curves of PTA‐1000 with different rotation speeds from 400 to 2500 rpm (Figure 4c) show an increased current density with increasing rotation speed. The similar observations were also found in other catalysts (Figure S16, Supporting Information). The valley peak at ≈0.73 V in the LSV curves of PTA‐1000 at the rotation rates of <1225 rpm may be originated from the short supply of O_2_ at low rotation speeds due to the fast depletion of O_2_ by PTA‐1000,^[^ ^22^ ^]^ indicating its high activity. The Koutecky–Levich (K–L) plots of all samples at different potentials (0.2–0.4 V) were acquired based on the LSV curves (Figure 4d; Figure S17, Supporting Information), from which the numbers of electron transferred (*n*) per O_2_ molecule were calculated (Figure 4e). PTA‐1000 is demonstrated to favor a 4e^−^ ORR process with an average *n* of 3.84, higher than those of PTA‐800 (2.41), 900 (3.18), 1100 (3.42), and TA‐1000 (2.09), only slightly lower than that of Pt/C and comparable with most reported defective carbon‐based catalysts (Table S4, Supporting Information).^[^ ^4^, ^21^ ^]^ The corresponding Tafel slope plots are shown in Figure 4f, the smallest slope of 74.2 mV decade^−1^ is observed for PTA‐1000 among all other compared catalysts, indicating the superior reaction kinetics. Moreover, the electrochemical impedance spectroscopy (EIS) measurements for various catalysts were conducted. The Nyquist plots (Figure S18, Supporting Information) show that PTA‐1000 possesses the smallest charge transfer resistance, indicating fast charge transfer at the electrode–electrolyte interface.^[^ ^23^ ^]^

The formation of peroxide species (H_2_O_2_) during the ORR process of all catalysts was also determined (Figure 4g; Figure S19, Supporting Information). The measured peroxide yield of PTA‐1000 is <15% in the potential range of −0.125 to 0.6 V, moderately higher than that of Pt/C catalyst (≈10%) but obviously lower than those of PTA‐800, 900, 1100, and TA‐1000. The average transfer number of PTA‐1000 was calculated to be >3.7 based on the ring and disk currents, comparable to Pt/C (≈3.8) and higher than other synthesized samples, which is consistent with the results of K–L plots. The observations further demonstrate a four‐electron pathway dominated ORR process catalyzed by PTA‐1000.

Apart from the catalytic activity, the stability and tolerance to methanol crossover effect are also two crucial parameters for ORR catalysts. The long‐time stability of PTA‐1000 was measured by a chronoamperometric response in O_2_‐saturated 0.1 m KOH solution at 1600 rpm (Figure 4h). After 20 000 s, the PTA‐1000 catalyst still retains 95.2% of the initial current, compared with 86.7% for Pt/C, indicating a better catalytic stability. Meanwhile, the methanol crossover was also examined by monitoring current responses in an O_2_‐saturated electrolyte with methanol injection at 500 s (Figure 4i). Almost no current density decay was observed for PTA‐1000 after adding methanol. However, a dramatic decrease with ≈46.2% in current density for Pt/C was recorded, suggesting a better tolerance to methanol for PTA‐1000.

The ORR performance under acidic conditions was also investigated in O_2_‐saturated 0.1 m HClO_4_ electrolyte. As shown in Figures S20, S21, and Table S5 (Supporting Information), the *E*_1/2_ of PTA‐1000 (0.66 V) was lower than that of the commercial Pt/C catalyst (0.77 V), indicating a good ORR catalytic activity in acidic electrolyte. The average electron transfer number of PTA‐1000 was calculated to be 3.80 by K–L equations, demonstrating an excellent ORR selectivity. The RRDE measurement of PTA‐1000 showed a low peroxide yield of below 14% and electron transfer number above 3.7, over the potential range of 0.102–0.6 V, further verifying the high selectivity. In addition, the smaller Tafel slope on PTA‐1000 (84.4 mV decade^−1^) than that of Pt/C (150.4 mV decade^−1^) revealed a superior reaction kinetics in acid solution. Collectively, the designed PTA‐1000 exhibits efficient ORR catalytic performances in both alkaline and acidic medium.

The remarkable ORR performance of PTA‐1000 may be attributed to be the following reasons. First, the highest contents of D1 and D2 type defects as active sites endow PTA‐1000 with a high intrinsic activity. Second, the abundant interlayer macropores with in plane micro‐mesopores promote the active site exposure and mass diffusion. Finally, the superstructure composed of interconnected carbon layers accelerates the electron transfer and boost the stability. The combination of these merits leads to the superior ORR performance of PTA‐1000 with excellent activity and stability.

## 3 Conclusion

In summary, we report the synthesis of superstructured macroporous carbon rods composed of defective graphitic carbon nanosheets by direct carbonization of crystalline poly tannic acid (PTA) rods as precursors. During carbonization process, PTA rods with a highly ordered lamellar structure trigger a spatially confined two‐step localized contraction, resulting in the sponge‐like macroporous carbon superstructure with large surface area, high porosity and abundant defects. Owing to these structural merits, the resultant defective microporous carbon superstructure demonstrates superior electrocatalytic performance with high activity and robust stability for oxygen reduction reaction. Our work has provided a new approach to synthesize macroporous carbon superstructures with distinctive structures and applications such as in electrocatalysis.

## 4 Experimental Section

### Synthesis of Carbon Superstructure

0.2 g of F127 was dissolved in 46 mL of water and 8 mL of ethanol at room temperature (20 °C). Then, 0.1 mL of ammonium hydroxide was added in the above solution. After stirring for 1 h, 0.2 g of tannic acid was added in the system and the mixture was stirred for another 24 h. The obtained mixture was transferred to an autoclave and hydrothermally treated for 24 h at 100 °C. The poly(tannic acid) rod‐like solid product was collected after centrifugation and washed with water for three times. The obtained product was dried at 60 °C in oven and denoted as PTA. The carbon material was obtained after carbonization of PTA at different temperatures for 3 h with a heating rate of 1 °C min^−1^ under nitrogen, denoted as PTA‐T (*T* = 800, 900, 1000, and 1100). For comparison, TA was directly carbonized into carbon at 1000 °C, resulting in TA‐1000.

## Conflict of Interest

The authors declare no conflict of interest.

## Supporting information

Supporting Information

## Data Availability

Research data are not shared.
