# Supplementary material for: Superstructured Macroporous Carbon Rods Composed of Defective Graphitic Nanosheets for Efficient Oxygen Reduction Reaction
Source: Adv Sci (Weinh). 2021 Jul 29;8(18):2100120. doi: 10.1002/advs.202100120 (PMC8456237; doi:10.1002/advs.202100120)
Supplement: Supplementary file 1 — Supporting Information [file ADVS-8-2100120-s001.pdf]

WILEY-VCH

© 2021 Wiley-VCH GmbH

ADVANCED SCIENCE
Open Access

Supporting Information

for *Adv. Sci.,* DOI: 10.1002/advs.202100120

Superstructured Macroporous Carbon Rods Composed of Defective Graphitic Nanosheets for Efficient Oxygen Reduction Reaction

Jing Wang, Yining Yao, Chaoqi Zhang, Qiang Sun, Dan Cheng, Xiaodan Huang, Jiayou Feng, Jingjing Wan, Jin Zou, Chao Liu, and Chengzhong Yu**

Supporting Information

Superstructured Macroporous Carbon Rods Composed of Defective Graphitic Nanosheets for Efficient Oxygen Reduction Reaction

Jing Wang, Yining Yao, Chaoqi Zhang, Qiang Sun, Dan Cheng, Xiaodan Huang, Jiayou Feng, Jingjing Wan, Jin Zou, Chao Liu, Chengzhong Yu**

Experimental Section

Chemicals: Tannic acid (99%, Adamas-beta), ammonium hydroxide (25-28 wt%) and anhydrous ethanol were purchased from Shanghai Titan Scientific Co., Ltd. Pluronic® F127, platinum on graphitized carbon (Pt/C, 20 wt%, a commercial catalyst) and Nafion (5 wt%) were purchased from Sigma-Aldrich. All chemicals are of analytical grade and used as received. Millipore deionized water was used in all experiments.

Characterization: Transmission electron microscopy (TEM) images were obtained with a JEM-2100F (JEOL) at 200 kV. Scanning electron microscopy (SEM) images were acquired by scanning electron microscope (HITACHI-S4800). N_2 sorption isotherms were measured using Micromeritics ASAP-2460 at liquid nitrogen temperature (-196 °C). X-ray diffraction (XRD) patterns were recorded using a Bruker D8 Advanced X-Ray Diffractometer with Cu Kα radiation (λ=0.154 nm). Fourier transform infrared (FT-IR) spectra of samples were obtained by using FT-IR-Nicolet IS-10 Thermo Fisher. The X-ray photoelectron spectra (XPS) of samples were characterized by ESCALAB 250xi with Al Kα radiation at 1486.6 eV. The Raman spectra were collected on Renishaw Invia Raman spectrometer at 532 nm. Thermogravimetric analysis (TGA) was conducted by SDT Q600 (TA Instruments) in N_2 with a ramp rate of 10 °C min^{-1}. MALDI-MS spectra of samples were performed on Autoflex MALDI-TOF/TOF (Bruker).

Electrochemical measurements: Electrochemical measurements (CHI 760E, CH Instrument, Shanghai), rotating disk electrode (RDE) and rotating ring-disk electrode (RRDE) (Pine Research Instrumentation, USA) characterizations were conducted in a standard three-electrode system. A graphite carbon rod served as the counter electrode, an Hg/HgO electrode (NaOH, 1.0 M) and Hg/Hg_2SO_4 (K_2SO_4, saturated) as the reference electrode, which was used in 0.1 M KOH and 0.1 M $HClO_4$, respectively, and a catalyst-modified glassy carbon electrode (5.61 mm in diameter, Pine) as the working electrode. To modify the glassy carbon electrode, 2 mg of catalysts were dispersed in 400 μL of alcohol solution containing 14 μL of Nafion solution (5 wt %) by sonication to form a homogeneous suspension. The obtained suspension was pipetted onto a polished glassy carbon electrode surface and dried at room temperature. The mass loading on the working electrode is 0.6 mg cm^{-2}. The commercially available Pt/C catalyst electrodes with the same mass loading (0.6 mg cm^{-2}) as working electrode was utilized for comparison.

In alkaline medium, the cyclic voltammetry (CV) tests of the samples in O_2-saturated 0.1 M KOH solution were performed in the potential range of -1.0 to 0.2 V (vs. Hg/HgO) with a scan rate of 5 mV s^{-1}. Linear sweep voltammograms (LSV) were acquired through the rotating disk electrode (RDE) technique in O_2-saturated 0.1 M KOH at a scan rate of 5 mV s^{-1} from -1.0 to 0.2 V (vs. Hg/HgO) under various rotation speeds (400-2500 rpm). In acid medium, all tests were studied in O_2-saturated 0.1 M $HClO_4$ at a scan rate of 5 mV s^{-1} from -0.6 to 0.4 V (vs. Hg/Hg_2SO_4). The electron transfer number (n) gained per O_2 involved in a typical ORR process was estimated by using the Koutecky-Levich (K-L) equations ((1)-(2)):

$$\frac{1}{j}=\frac{1}{j_k}+\frac{1}{j_L}=\frac{1}{j_k}+\frac{1}{B\omega^{1/2}} \quad (1)$$

$$\mathrm{n}=\frac{B}{0.62FC_{O_2}(D_{O_2})^{2/3}(v)^{-1/6}} \quad (2)$$

Where j_k is the kinetic current, B can be determined from the slope of the K-L plots. F is the Faraday constant, D_{O2} is the diffusion coefficient of O_2, v is the kinetic viscosity of the electrolyte and C_{O2} is the bulk concentration of O_2.

For the RRDE measurements, the ring electrode potential was set as 0.5 V (vs. Hg/HgO). The hydrogen peroxide yield and electron transfer number (n) were calculated by the following equations ((3)-(4)):

$$H_2O_2\% = \frac{200I_r/N}{I_d+I_r/N} \quad (3)$$

$$\mathrm{n} = \frac{4I_d}{I_d+I_r/N} \quad (4)$$

where I_d and I_r are the disk and ring currents, respectively, and N (0.37) is the current collection coefficient of Pt ring.

The stability and methanol tolerance tests were performed at a potential of -0.423 V (vs. Hg/HgO) in O_2-saturated 0.1 M KOH solution for the chronoamperometry at room temperature with the working electrode rotating at 1600 rpm. The addition of 3 wt% methanol in an O_2-saturated 0.1 M KOH electrolyte was used to examine the methanol tolerance of catalysts.

All the potentials were calibrated with a reversible hydrogen electrode (RHE) (Figure S14). The calibration was performed in the high purity hydrogen saturated electrolyte in a standard three-electrode system with Pt wires as the working and counter electrodes, and the Hg/HgO and Hg/Hg_2SO_4 electrode as the reference electrode under basic and acidic conditions, respectively. Linear scanning voltammetry (LSV) was then performed at a scan rate of 5 mV s^{-1}, and the potential at the current crossed zero was taken as the thermodynamic potential for the hydrogen electrode reactions.

Figures and Tables

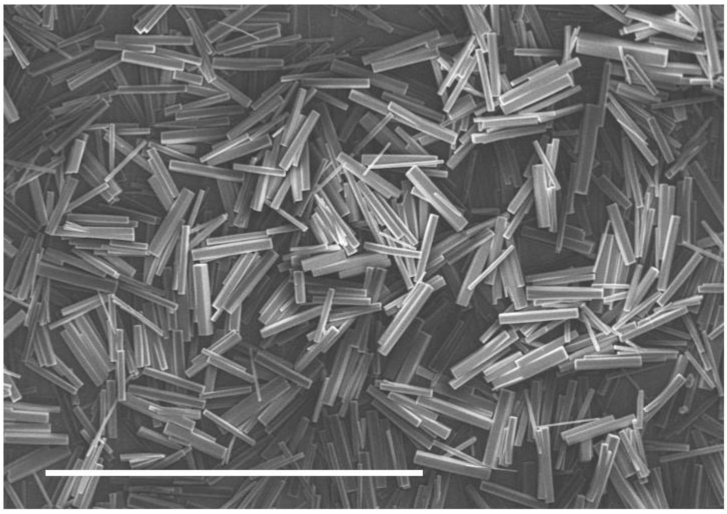

Figure S1. Low magnification SEM image of PTA. The scale bar is 100 μm.

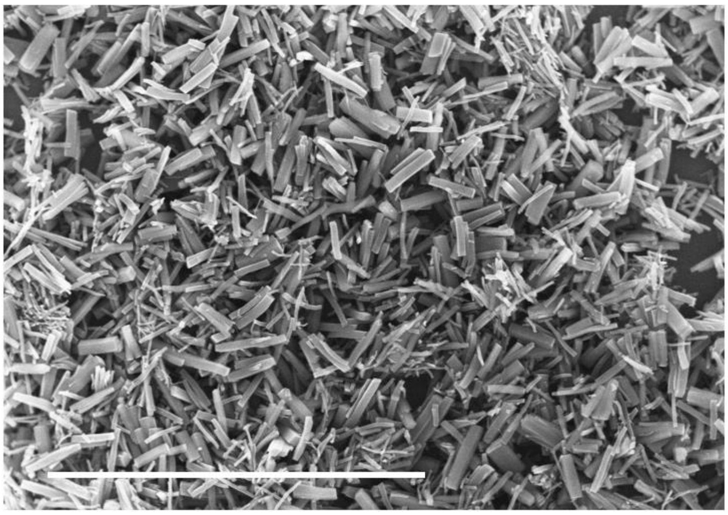

Figure S2. Low magnification SEM image of PTA-1000. The scale bar is 100 μm.

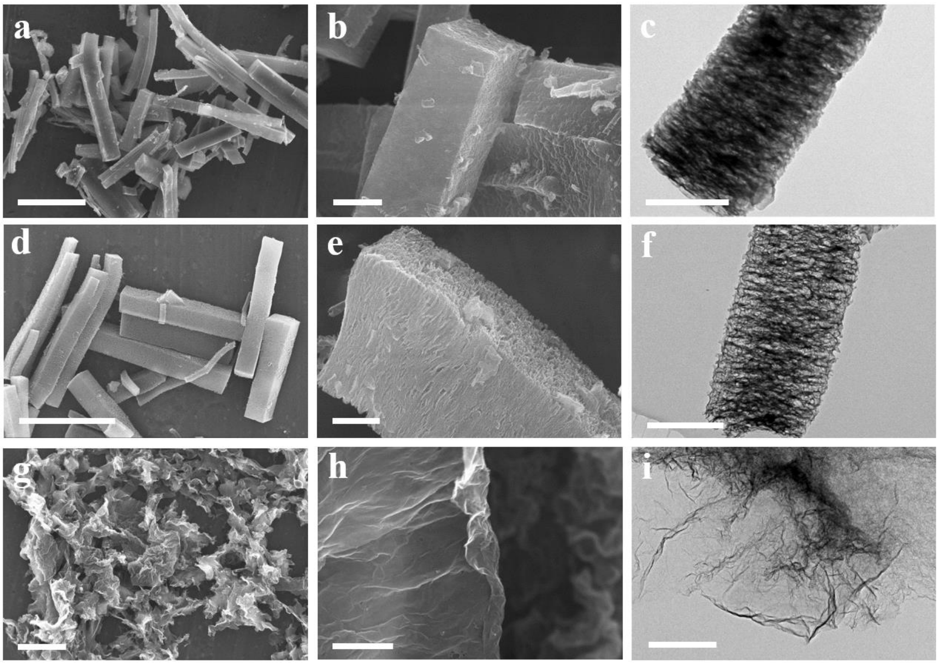

Figure S3. SEM (a, b, d, e, g, h) and TEM (c, f, i) images of PTA-800 (a-c), PTA-900 (d-f) and PTA-1100 (g-i). Scale bars are (a, d, g) 10 μm, (b, c, f) 1 μm, (e, h, i) 500 nm.

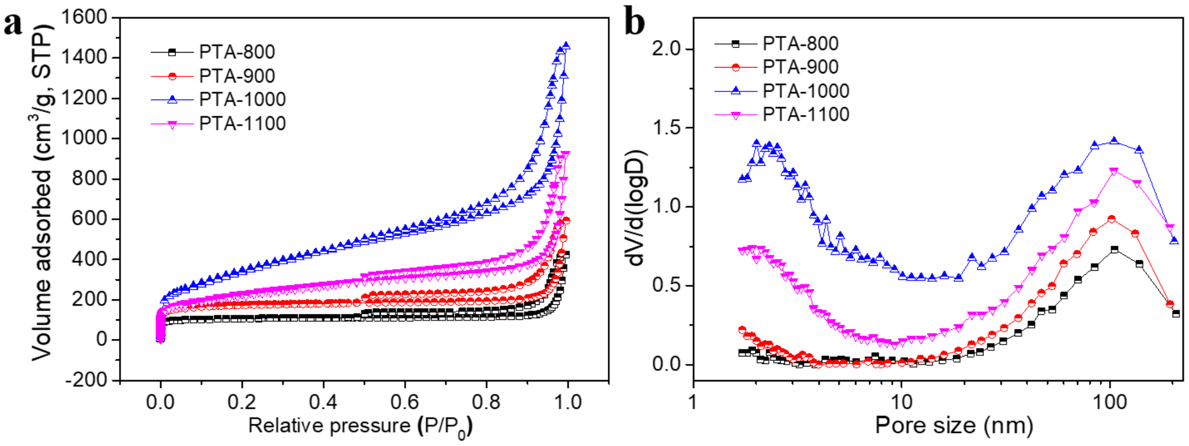

Figure S4. (a) N_2 sorption isotherms, (b) pore size distribution curves of PTA-800, 900, 1000 and 1100.

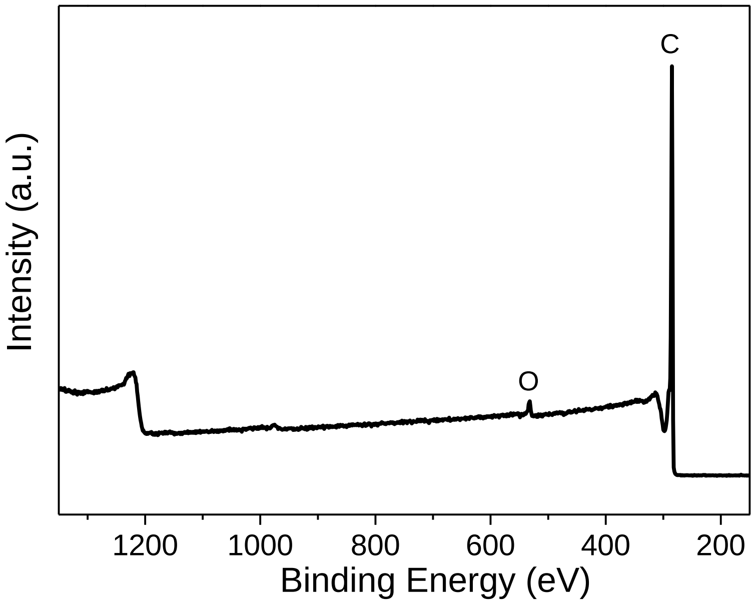

Figure S5. XPS survey spectrum of PTA-1000

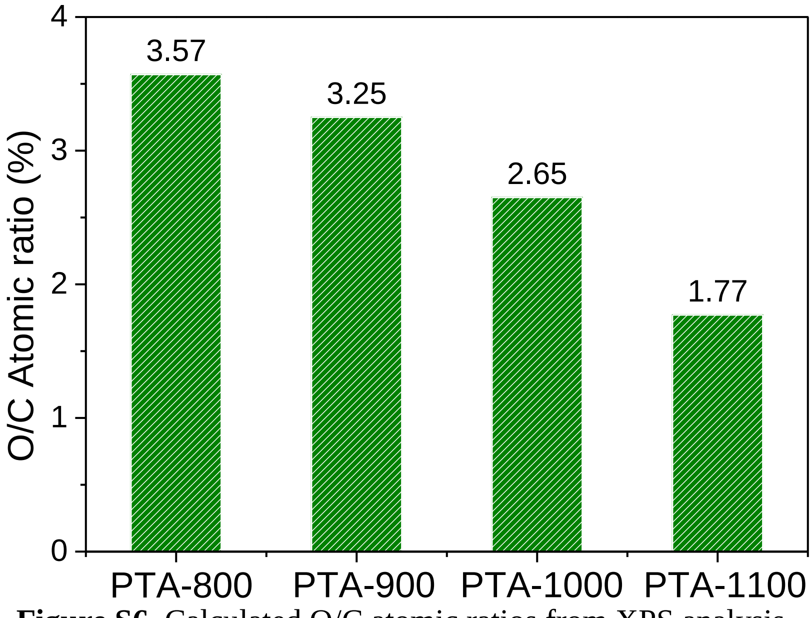

Figure S6. Calculated O/C atomic ratios from XPS analysis.

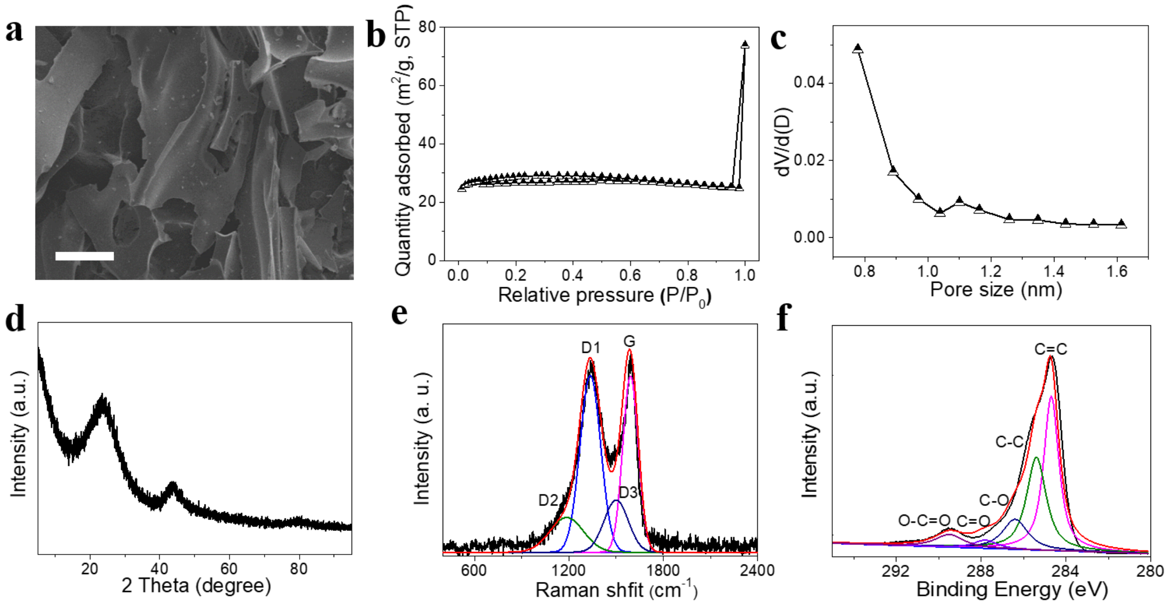

Figure S7 (a) SEM image, (b) N_2 sorption isotherm, (c) pore size distribution curve, (d) XRD pattern, (e) Raman spectrum, (f) XPS spectrum of C1s of TA-1000. The scale bar is 10 μm in (a).

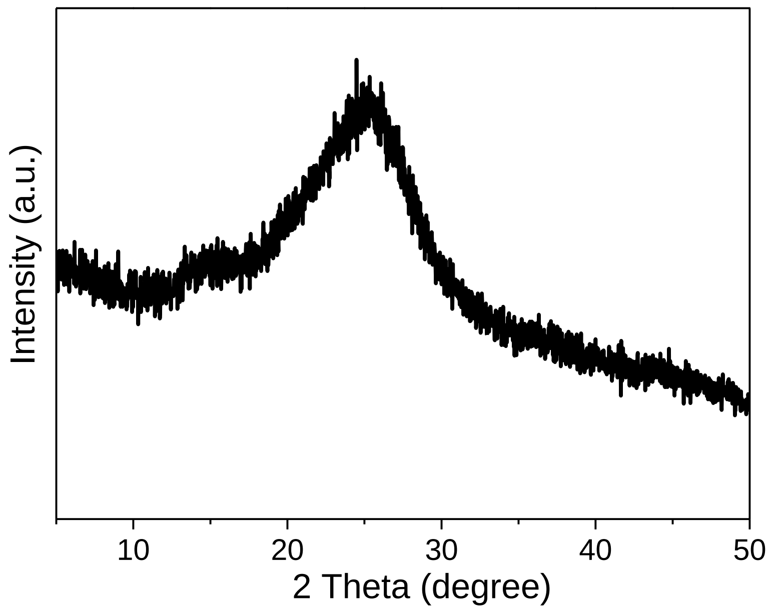

Figure S8. XRD pattern of TA.

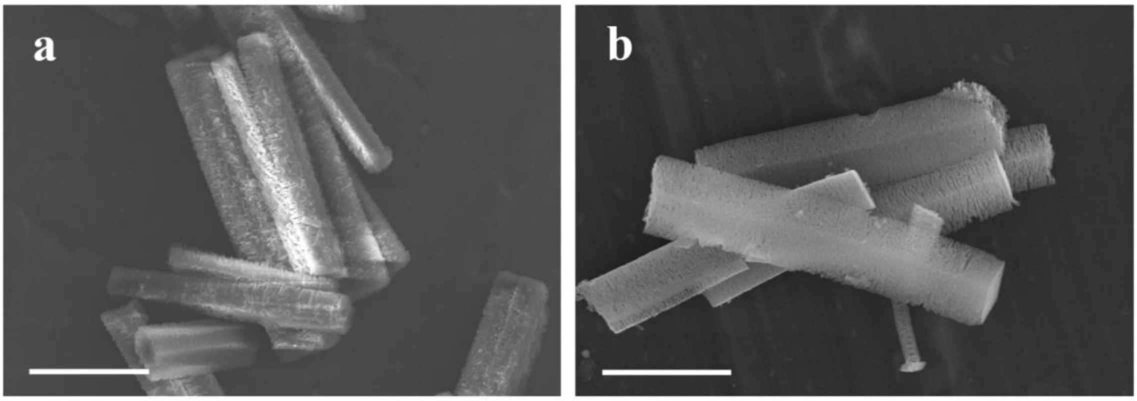

Figure S9. SEM images of (a) PTA-250 and (b) PTA-450. The scale bar is 5 μm in (a) and (b)

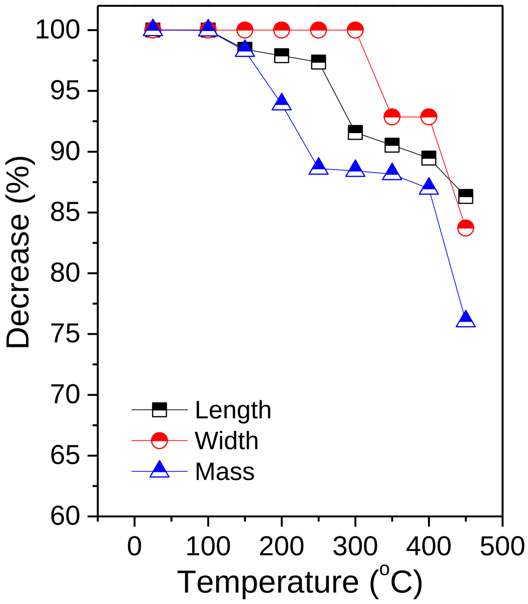

Figure S10. Change of length, width and weight of PTA during heat treatment process.

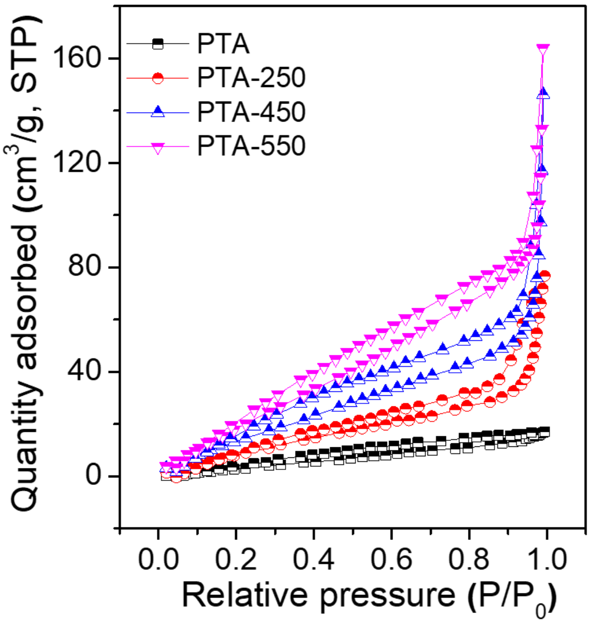

Figure S11. N_2 sorption isotherms of PTA and PTA-250, 450 and 550.

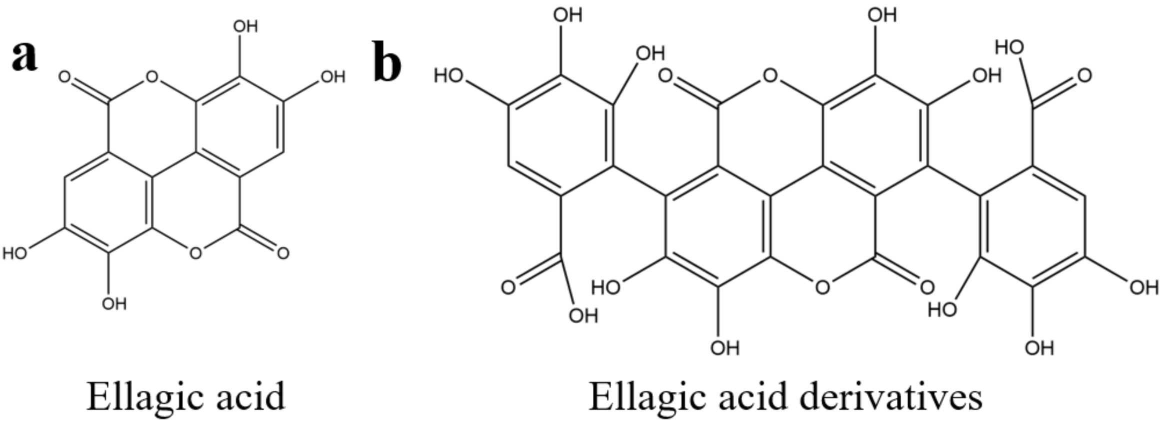

Figure S12. Chemical structures of possible molecules in PTA.

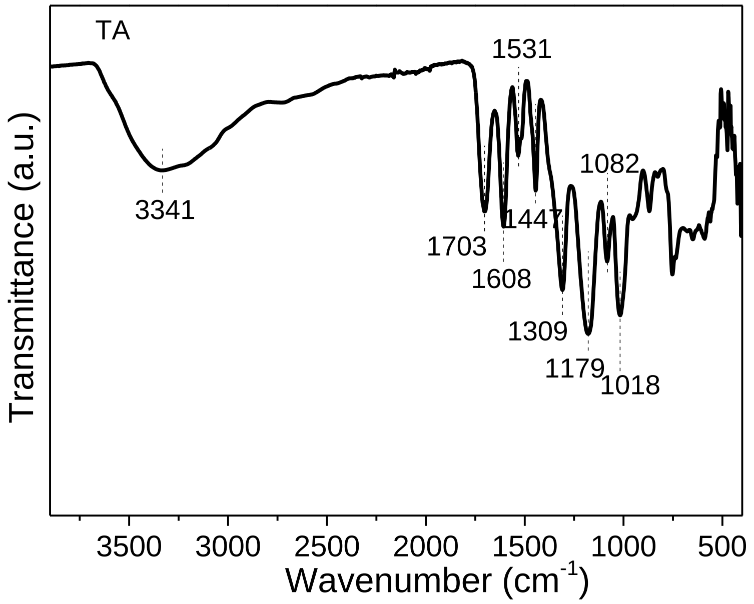

Figure S13. FTIR spectrum of TA. Typical groups of phenol O-H (3341 cm^{-1}), C=O of ester (1703 cm^{-1}), aromatic ring (1608, 1531, 1447 cm^{-1}), sub-benzene (1179 cm^{-1}) and C-O-C (1082, 1018 cm^{-1}) are found.

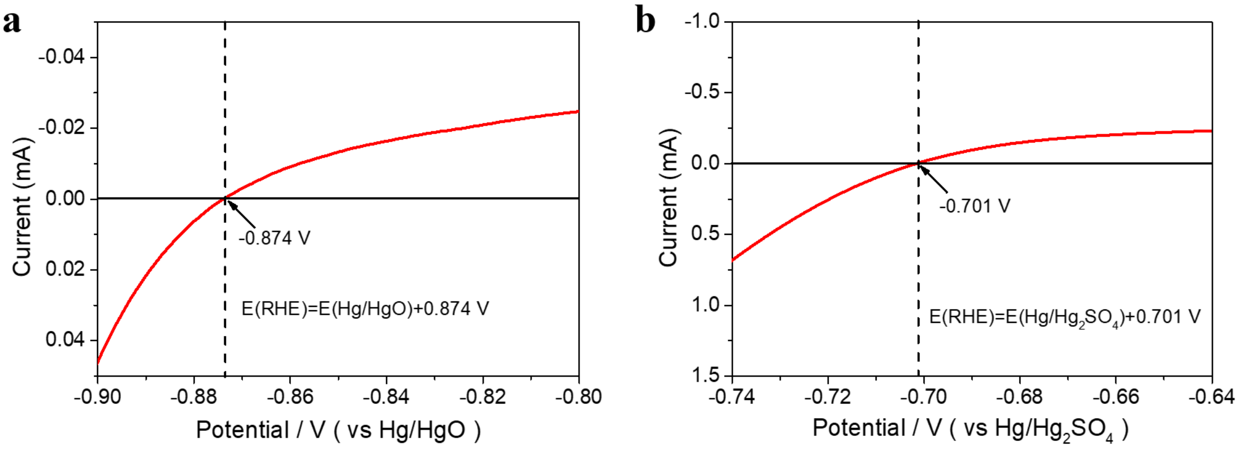

Figure S14. Calibration to reversible hydrogen electrode (RHE) in (a) 0.1 M KOH, (b) 0.1 M $HClO_4$

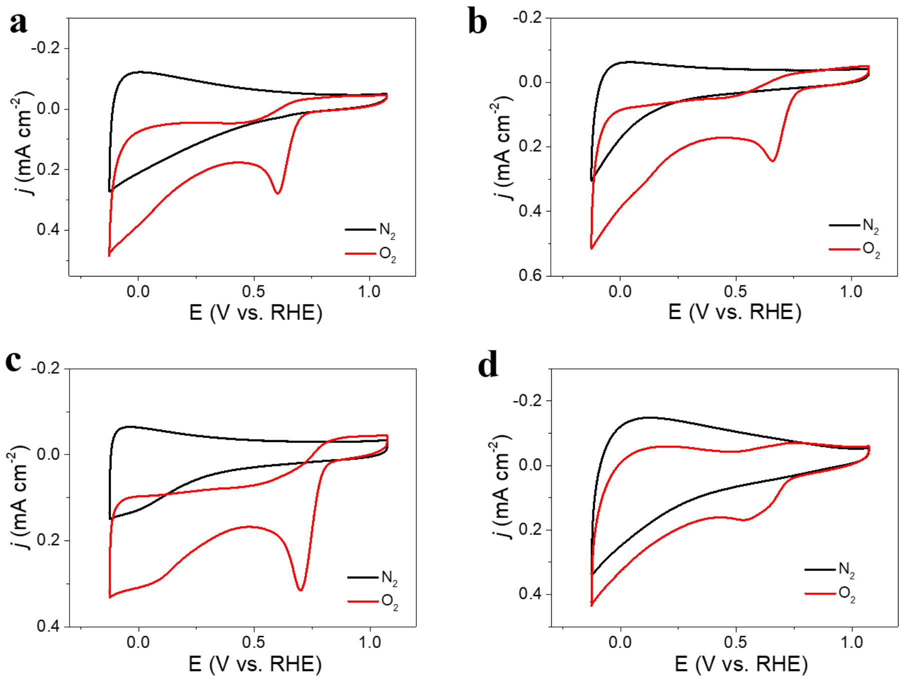

Figure S15. CV curves of (a) PTA-800, (b) PTA-900, (c) PTA-1100 and (d) TA-1000 in O_2 or N_2-saturated 0.1 M KOH solution with a scan rate of 5 mV s^{-1}.

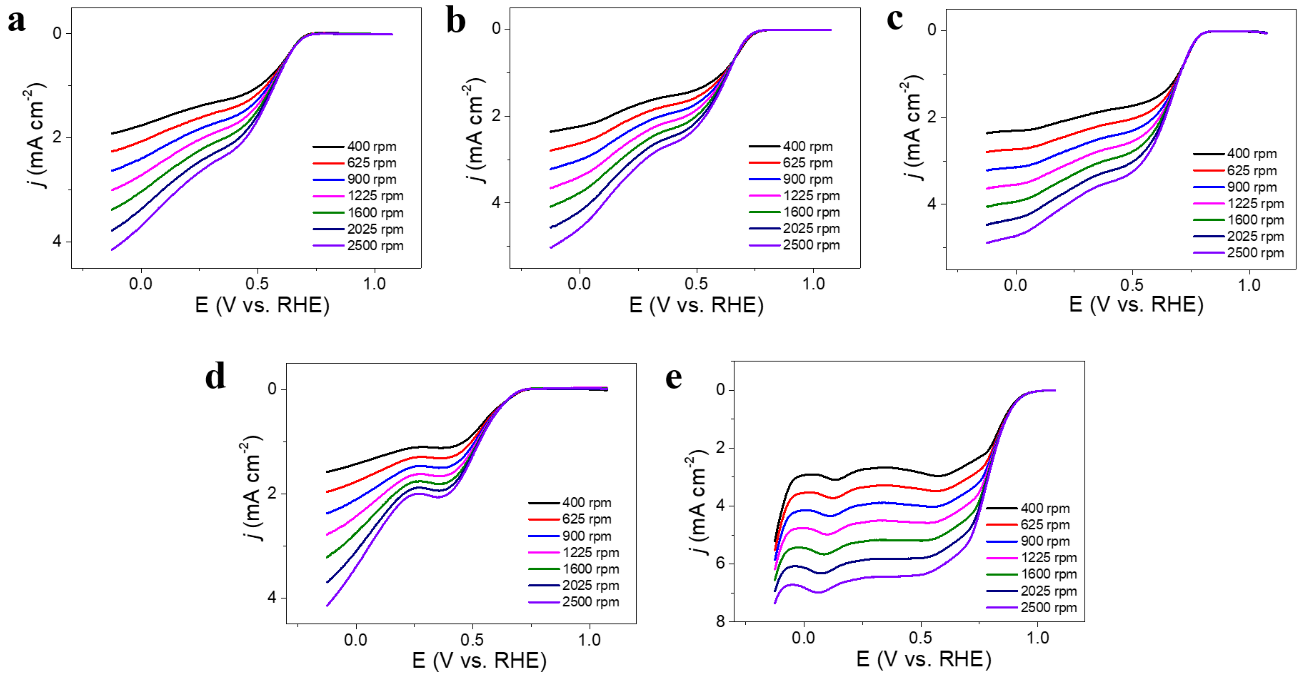

Figure S16. LSV curves of (a) PTA-800, (b) PTA-900, (c) PTA-1100, (d) TA-1000 and (e) Pt/C in O_2 -saturated 0.1 M KOH solution at different rotation rates.

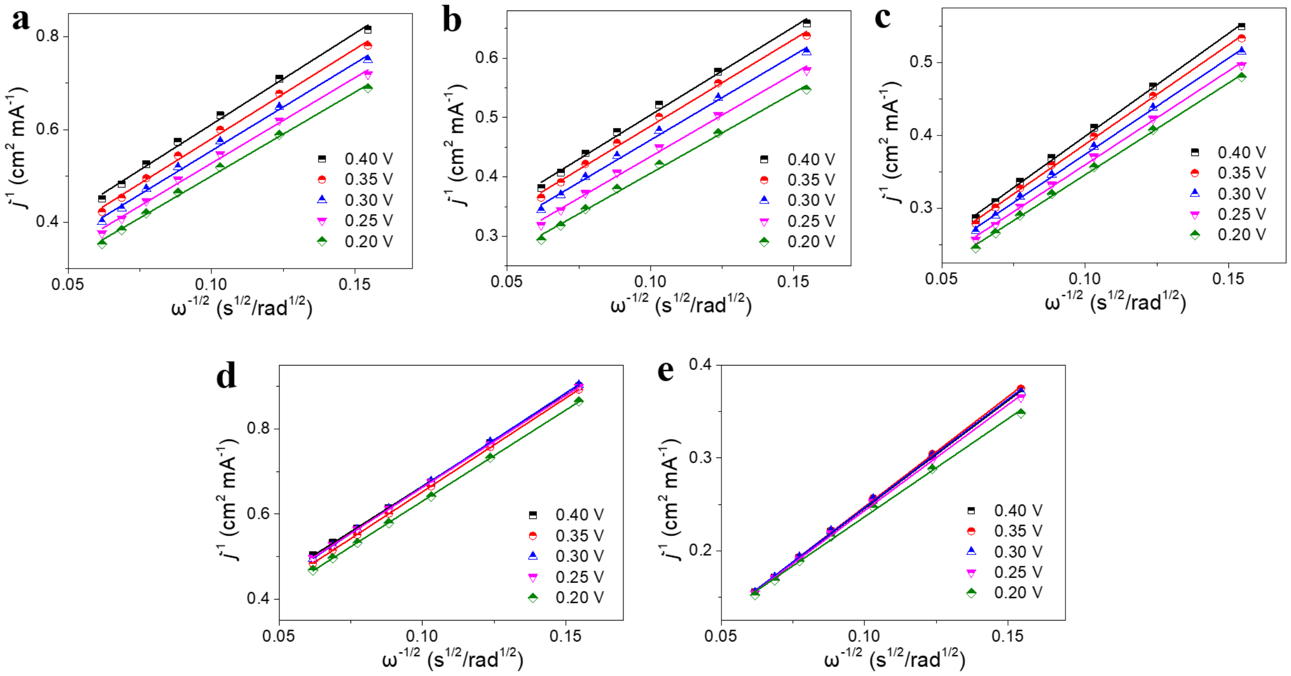

Figure S17. Koutecky-Levich plots of (a) PTA-800, (b) PTA-900, (c) PTA-1100, (d) TA-1000 and (e) Pt/C derived from the polarization curves at different electrode potentials.

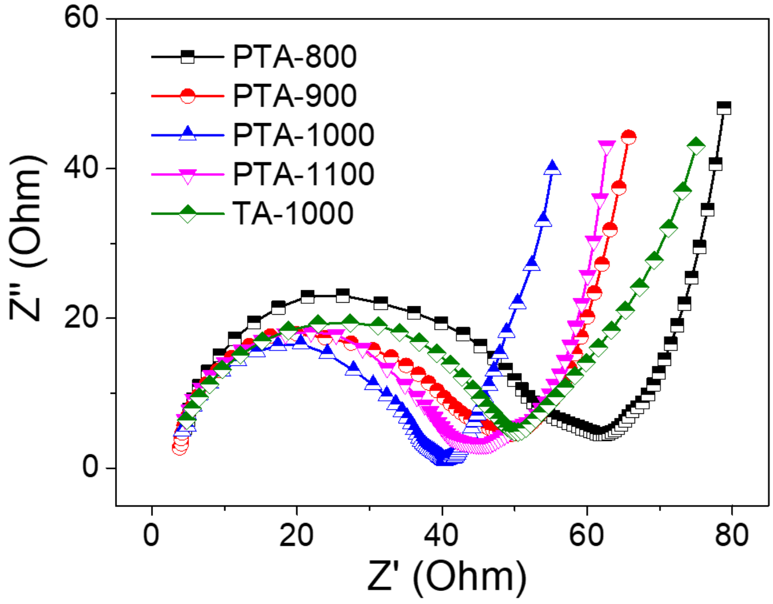

Figure S18. Electrochemical impedance spectroscopy (EIS) spectra of PTA-800, PTA-900, PTA-1000, PTA-1100 and TA-1000.

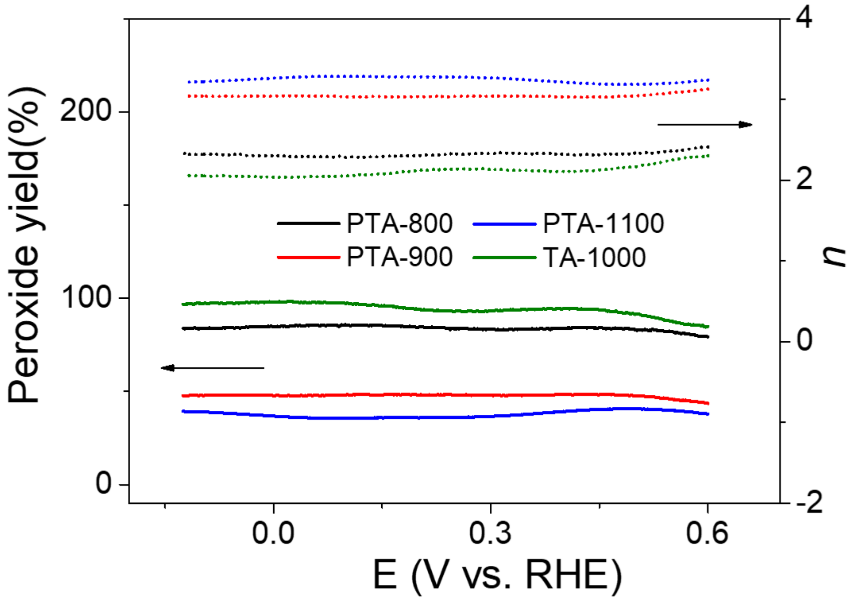

Figure S19. Percentage of peroxide species (solid lines) and the electron transfer number (n) (dotted lines) of PTA-800, PTA-900, PTA-1100 and TA-1000 at different potentials (calculated from the corresponding RRDE data) in O_2 saturated 0.1 M KOH solution at 1600 rpm.

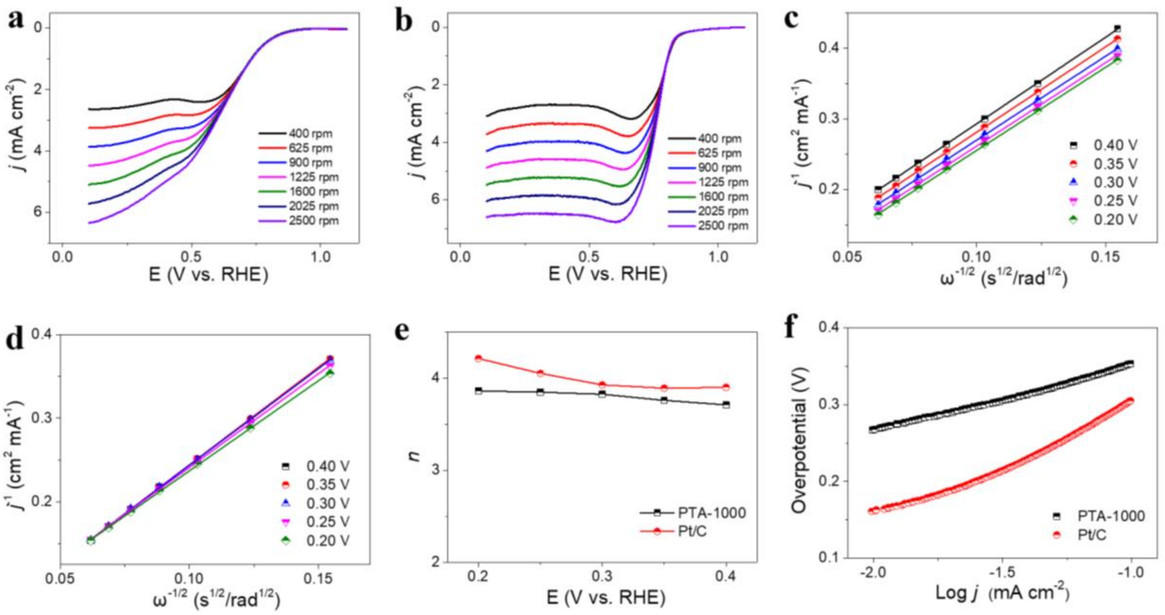

Figure S20. LSV curves of (a) PTA-1000 and (b) commercial Pt/C in O_2 saturated 0.1 M $HClO_4$ solution at different rotation rates. Koutecky-Levich plots of (c) PTA-1000 and (d) commercial Pt/C derived from the LSV curves in (a) and (b) at different potentials, respectively. (e) Electron transfer number (*n*) calculated from K-L equations at different potentials of test samples. (f) Tafel plots of test samples.

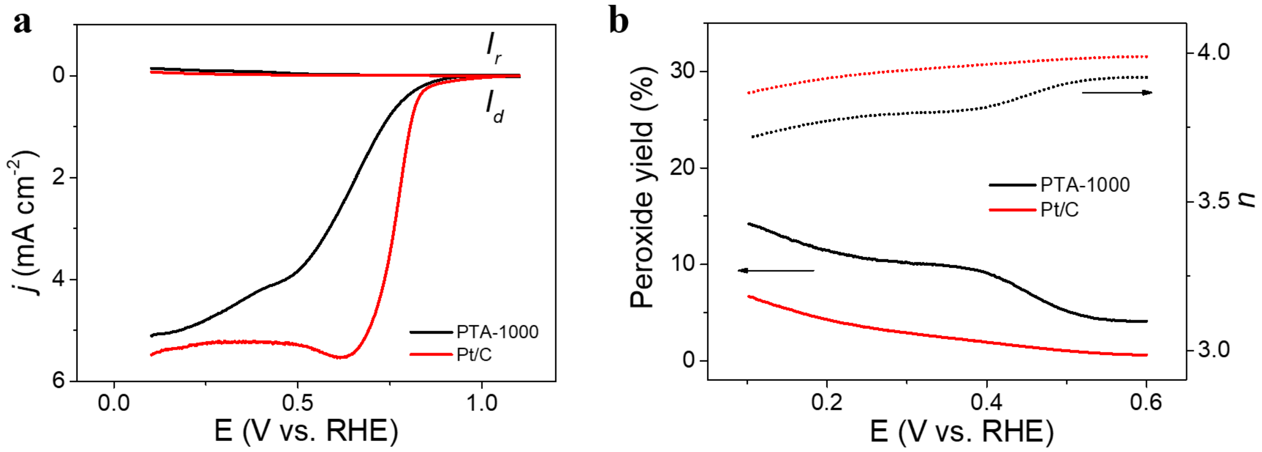

Figure S21. (a) RRDE spectra of the PTA-1000 and commercial Pt/C in O_2 saturated 0.1 M $HClO_4$ solution at 1600 rpm. (b) Percentage of peroxide species (solid lines) and the electron transfer number (n) (dotted lines) of PTA-1000 and commercial Pt/C at different potentials (calculated from the corresponding RRDE data).

Table S1. Textural properties of synthesized samples.

Sample	S_{BET} (m^2/g)[a]	V_{micro} (cm^3/g)[b]	V_{meso} (cm^3/g)[c]	V_{Total} (cm^3/g)[d]
PTA-800	335	0.15	0.10	0.65
PTA-900	558	0.23	0.19	0.92
PTA-1000	1256	0.34	1.04	2.25
PTA-1100	800	0.24	0.49	1.43
TA-1000	89	0.04	0.001	0.10

a)The specific surface area; b)Micropore volume; c)Mesopore volume; d)Total pore volume

Table S2. The ratios of I_{D1}/I_G, I_{D2}/I_G and I_{D3}/I_G

Sample	I_{D1}/I_G	I_{D2}/I_G	I_{D3}/I_G
PTA-800	1.08	0.35	0.40
PTA-900	1.32	0.42	0.45
PTA-1000	1.98	0.58	0.60
PTA-1100	1.67	0.49	0.53
TA-1000	1.34	0.43	0.46

Table S3. ORR electrocatalysis properties of samples in O_2 saturated 0.1 M KOH solution

Sample	E_{onset} (V)[a)]	$E_{1/2}$ (V)[b)]	n[c)]	b (mv dec^{-1})[d)]
PTA-800	0.73	0.57	2.41	125.9
PTA-900	0.79	0.63	3.18	103.1
PTA-1000	0.96	0.78	3.84	74.2
PTA-1100	0.83	0.68	3.42	86.2
TA-1000	0.72	0.53	2.09	157.7
Pt/C	1.00	0.80	3.98	97.7

a) Onset potential; b) Half wave potential; c) The average transferred electron number calculate from K-L equations between potentials of 0.2-0.4 V; d) Tafel slope.

Table S4. Comparison of ORR performance with recently reported electrocatalysts in O_2 saturated 0.1 M KOH solution

Catalysts	E_{onset} (vs RHE) /V	$E_{1/2}$ (vs RHE) /V	n	Ref.
D-MNS-A	0.87	0.75	3.7	[21]
Defective graphene	0.91	0.76	3.87	[4b]
PC-I8-950	0.93	0.80	3.6	[4p]
D-CM	0.95	0.81	4	[4e]
Pt/C	1.00	0.80	3.98	This work
PTA-1000	0.96	0.78	3.84	This work

Table S5. ORR electrocatalysis properties of samples in O_2 saturated 0.1 M $HClO_4$ solution

Sample	E_{onset} (V)[a)]	$E_{1/2}$ (V)[b)]	n[c)]	b (mv dec^{-1})[d)]
PTA-1000	0.93	0.66	3.80	84.4
Pt/C	0.98	0.77	3.99	150.4

[a)] Onset potential; [b)] Half wave potential; [c)] The average transferred electron number calculate from K-L equations between potentials of 0.2-0.4 V; [d)] Tafel slope
